# PPAR*α*- and DEHP-Induced Cancers

**DOI:** 10.1155/2008/759716

**Published:** 2008-08-31

**Authors:** Yuki Ito, Tamie Nakajima

**Affiliations:** Department of Occupational and Environmental Health, Nagoya University Graduate School of Medicine, 65 Tsurumai-cho, Showa-ku, Nagoya 466-8550, Japan

## Abstract

Di(2-ethylhexyl)phthalate (DEHP) is a widely used plasticizer and a potentially nongenotoxic carcinogen. Its mechanism had been earlier proposed based on peroxisome proliferator-activated receptor *α* (PPAR*α*) because metabolites of DEHP are agonists. However, recent evidence also suggests the involvement of non-PPAR*α* multiple pathway in DEHP-induced carcinogenesis. Since there are differences in the function and constitutive expression of PPAR*α* among rodents and humans, species differences are also thought to exist in the carcinogenesis. However, species differences were also seen in the lipase activity involved in the first step of the DEHP metabolism, which should be considered in DEHP-induced carcinogenesis. Taken together, it is very difficult to extrapolate the results from rodents to humans in the case of DEHP carcinogenicity. However, PPAR*α*-null mice or mice with human PPAR*α* gene have been developed, which may lend support to make such a difficult extrapolation. Overall, further mechanical study on DEHP-induced carcinogenicity is warranted using these mice.

## 1. INTRODUCTION

Di(2-ethylhexyl)phthalate (DEHP) a plasticizer around the world, suggesting that many people come across this chemical every day. Animal studies showed that this chemical is a nongenotoxic carcinogen. Metabolites of DEHP, mono- and dicarboxylic acids, transactivate peroxizome proliferator-activated receptor *α* (PPAR*α*), which has been thought to result in nongenotoxic carcinogenesis [1, 2]. However, the latest studies also showed the involvement of non-PPAR*α* pathways; multiple pathways might be involved in the pathway of DEHP-induced carcinogenicity [3]. There are species differences in the functional activation or constitutive expression of rodent and human PPAR*α*, and that in humans is thought to be less active and expressive than those of rodents. Recently, inflammation-related carcinogenesis has drawn attention [4, 5]. PPAR*α* is involved not only in the induction of target genes such as *β*-oxidation enzymes of fatty acids but also in anti-inflammation signaling [6, 7], suggesting that PPAR*α* also may protect against carcinogenesis. Species differences in lipase activity (DEHP-metabolizing enzyme) among mice, rats, and marmosets have been also reported recently [8], suggesting that this kinetic difference should be considered in the species differences in DEHP-induced carcinogenesis. In this review, we focused on DEHP-induced hepatic carcinogenesis in relation to PPAR*α*-dependent and PPAR*α*-independent pathways, and discussed the science policy.

## 2. PPARs

PPARs are involved in a member of the nuclear hormone receptor superfamily, and consist of three subunits: PPAR*α*, PPAR*β*/*δ*, and PPAR*γ* [9]. These three isoforms have been identified at the organ-specific level. In the respective organ, PPARs function as transcription factors through the classic ligand-dependent nuclear hormone receptor mechanism. Upon binding to their ligands, PPARs undergo conformational changes that allow corepressor release [10]. The PPAR-ligand complex binds to direct repeat 1 elements or peroxisome proliferator response elements (PPREs), usually located upstream of the target genes, which results in the induction of fatty acid transport and metabolism, glucose metabolism, and also elicitation of anti-inflammatory effects [6, 11].

As one of the three isoforms, PPAR*α* is mainly expressed in organs that are critical in fatty acid catabolism, such as liver, heart, and kidney [7]. Thus, this nuclear receptor is primarily involved in the regulation of fatty acid metabolism. In addition to this function, PPAR*α* also has various functions including the promotion of gluconeogenesis, lipogenesis, ketogenesis, and anti-inflammatory effects [6].

## 3. PPAR*α* LIGANDS

The ligands of PPAR*α* represent a diverse group of chemicals including not only endogenous ligands but also exogenous synthetic ligands with a high likelihood of clinical, occupational, and environmental exposure of humans to chemicals [1, 12]. The primary endogenous ligands are fatty acids, mainly the 18–20 carbon polyunsaturated fatty acids and eicosanoids [7, 13–17]. As exogenous ligands, fibrates and thiazolidinediones are involved. Additionally, the general population is exposed to environmental chemicals such as plasticizers (e.g., phthalates), solvents (e.g., tetrachloroethylene and trichloroethylene), perfluorooctanoic acid and herbicides (e.g., 2, 4-dichlorophenoxyacetic acid, diclofop-methyl, haloxyfop, lactofen, and oxidiazon).

Of these ligands, the toxicity of DEHP is well established in relation to PPAR*α*. This chemical is used as a plasticizer to improve the plasticity and elasticity of polyvinyl chloride products that have become ubiquitous in our daily living. These products are widely used in building materials, wallpaper and flooring, wire covering, vinyl sheeting for agriculture, food packages, and medical devices such as intravenous and hemodialysis tubing and blood bags. The recent production of DEHP in Japan has approached 14 000 tons per year, which accounts for about 54% of all plasticizers used [11]. It is noted that mono- and dicarboxylic acid metabolites of DEHP, not DEHP itself, act as ligands for PPAR*α* [18] and have potentially adverse effects on liver, kidney, heart, and reproductive organs though monocarboxylic acid, mono(2-ethylhexyl) phthalate (MEHP), also binds to PPAR*γ* [18].

## 4. SPECIES DIFFERENCES IN PPAR*α*

Since there are species differences in the toxicity of PPAR*α* agonists, the expression levels or functions of the receptor are thought to be different among species. Several explanations for the species differences in response to the ligands have been suggested [19, 20]. One of the major factors was considered to be due to differences in the levels of PPAR*α* expression [21, 22] although other possibilities include differences in ligand affinity between rodent and human PPAR*α*, differences in cellular context of PPAR*α* expression, and those in PPRE sequences found upstream of critical target genes [23, 24]. Indeed, PPAR*α* expression in humans is about 1/10 times less than that in rodents [25]. In addition, micro-RNA expression regulated by PPAR*α* has been recently reported to be changed in wild-type mice, but not in mice with human PPAR*α* gene [26]; Wy-14,643 inhibited a micro-RNA let-7C which is involved in suppression of tumorigenesis in wild-type mice, but neither in PPAR*α*-null mice nor in mice with human PPAR*α* gene. Mice with human PPAR*α* gene are resistant to hepatocellular proliferation though they respond to Wy-14,643 in *β*-oxidation and serum triglycerides [27]. These results suggest that the function of the PPAR*α* signaling in liver proliferation and tumorigenesis by the chemical exposure is not always similar in mice and humans.

In regard to the species differences in the PPREs, the lack of acyl CoA oxidase (ACO) induction in studies on liver biopsies from humans treated with hypolipidemic drugs or primary human hepatocytes treated with Wy-14,643 may be attributable to an inactive functional PPRE since the sequence of a PPRE for the ACO gene from a small number of human liver biopsy samples was found to be different from that of the rats [28]. However, Reddy remarked at a panel discussion that, although the sequence of ACO gene promoter in the mouse was also different from that in the rat, both rodents are responsive to some peroxisome proliferators in ACO induction [20]. In addition, differences in the ability of rodents and human PPAR to recognize and bind PPRE are unlikely since the DNA binding domains of the human and rodent PPAR*α* are 100% homologous [29, 30]. Though characterized from only a limited number of individuals, the prevalence in the population of defective PPAR alleles cannot be determined at this point [31]. The species difference in the sequence of PPRE may not be involved in the difference in response to ligands between rodents and humans.

In addition to the lower expression levels of PPAR*α* in human, there was a truncated, inactive form of PPAR*α* in human liver, suggesting that the expression of full-length functional PPAR*α* was very low. These inactive forms of PPAR*α* may be insufficient to bind PPRE because PPREs may be occupied in vivo by other nuclear receptors that bind to similar sequences, thus affecting responsiveness to ligands [25].

## 5. SPECIES DIFFERENCES IN DEHP METABOLISM

In addition to the species differences in PPAR*α* functions or expression levels, we should also be mindful of the importance of those in the metabolism of DEHP between rodents and humans. DEHP absorbed in the body is first metabolized by the catalytic action of lipase to produce MEHP and 2-ethylhexanol (2-EH) [32]. Some MEHP is then conjugated with UDP-glucuronide by UDP-glucuronosyltransferase (UGT) and excreted in the urine. The remaining MEHP is excreted directly in the urine or is oxidized by cytochrome P450 4A, then further oxidized by alcohol dehydrogenase (ADH) or aldehyde dehydrogenase (ALDH) to dicarboxylic acid or ketones. 2-EH is metabolized mainly to carboxylic acid (mainly 2-ethylhexanoic acid (2HEA)) via 2-ethylhexanal by catalytic action of ADH and ALDH. Thus, lipase may be an essential enzyme to regulate the DEHP metabolism; knowing the species difference in the lipase activity may be an important tool to clarify the species difference in metabolism.

**Figure 1:**
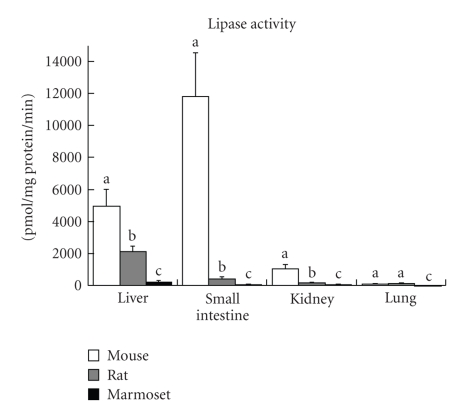
Species differences in lipase activities (pmol/mg protein in microsomal fragment/min) using hepatic microsomes in liver, small intestine, kidney, and lung from mice, rats, and marmosets. Lipase activity was measured by GC/MS. Substrate concentration (DEHP) used was 1 mM. Each white bar (6 mice), grey bar (5 rats), or black bar (5 marmosets) represents the mean ± standard deviations. Lipase activity was not detected in marmoset lung (under 1 pmol/mg protein/min). Comparisons were made using analysis of variance and the Tukey-Kramer HSD post hoc test. A logarithmic transformation was applied to lipase activities in microsome samples from the small intestine and kidneys before Tukey-Kramer analysis. Different letters (a, b, c) on the top of each bar in each organ indicate that they are significantly different from each other (*P* < .05).

**Figure 2:**
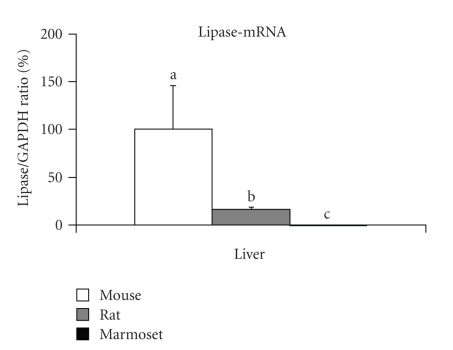
Lipase-mRNA levels in mice, rats, and marmosets. Each mRNA level was measured by real-time quantitative PCR and normalized to the GAPDH-mRNA level in the same preparation. Mouse liver mean was assigned a value of 100. Figures represent mean ± SD from 6 from mice and 5 from rats and marmosets. Comparisons were made using analysis of variance and the Tukey-Kramer HSD post hoc test. Different letters (a, b, c) on the top of each bar in each organ indicate that they are significantly different from each other (*P* < .05).

Recently, the activities of lipase, UGT, ADH, and ALDH for DEHP metabolism in several organs were measured and compared among mice, rats, and marmosets [8]. Marmosets were used as a reference to human. Clear-cut species differences were seen in the activities of the four enzymes involved in the DEHP metabolism among mice, rats, and marmosets. The most prominent difference was observed in the lipase activity with an almost 148- to 357-fold difference between the highest activity in mice and the lowest in marmosets (Figure 1). These differences were comparable to those in the kinetic parameter, *Vmax.* These results suggest that the constitutive levels of lipase were greater in the mice and rats than in marmosets. Indeed, lipase-mRNA levels in livers from mice or rats were much higher than those in marmoset (Figure 2). Thus, concentrations of MEHPs (ligands to PPAR*α*) in the body were higher in mice or rats than in marmosets when the same dose of DEHP was administered [33].

Besides species differences in the constitutive levels of lipase, *Km* values of DEHP for lipase of marmosets were much higher than in rats or mice, suggesting the species differences in the DEHP affinity for lipase; the affinity of DEHP for lipase in the marmosets may be lower than that of mice or rats. The affinity in human may be even lower than that in primates; cumulative ^14^C excretion in urine of African green monkey following bolus injection of ^14^C-DEHP leached into autologous plasma occurred earlier than in human [34].

## 6. MECHANISM OF DEHP-INDUCED CANCER

DEHP causes tumors, especially in liver when chronically administered to rats and mice [35–39], similar to the other peroxisome proliferators such as Wy-14643. Table 1 shows that DEHP induces hepatic tumors in mice and rats. From the viewpoint of percentage in feed, the lowest-observed effect-level (LOEL) of DEHP carcinogenicity in the rat was 0.6%, and the no-observed effect-level (NOEL) was 0.1% [2]. In the mouse, the corresponding values may be 0.05% for LOEL and 0.01% for NOEL because the study in which male mice were exposed to 0.05% DEHP for 78 weeks exhibited a significant increase in the hepatic tumor incidence rate compared with controls, but not when exposed to 0.01% DEHP [40].

DEHP also has potential for carcinogenesis in other organs; pancreatic acinar cell adenoma and mononuclear cell leukemia incidences were significantly increased in male F344 rat but not in F344 female rat and B6C3F1 mouse of both sexes after DEHP exposure [35, 36, 44]. The reason why these cancers are not observed in female rat has not been identified.

Chronic treatment with PPAR*α* agonist results in an increased incidence of liver tumors which were thought to have occurred through a PPAR*α*-mediated mechanism as revealed by the resistance of PPAR*α*-null mice to liver cancer induced by Wy-14,643 exposure for 11 months [46]. All the wild-type mice fed with 0.1% Wy-14643 diet for 11 months had multiple hepatocellular neoplasms, including adenomas and carcinomas, while thePPAR*α*-null mice fed with the 0.1% Wy-14643 diet for the same duration were unaffected. Ward et al. [47] reported that exposure for only six months to 12 000 ppm DEHP caused induction of peroxisomal enzymes, liver enlargement, and histopathological increases in eosinophil counts and peroxisomes in the cytoplasm of wild-type mice, while there were no such toxic findings in the liver of PPAR*α*-null mice. Thus, DEHP-derived carcinogenicity was thought to be mediated by PPAR*α*, similar to Wy-14,643, and DEHP was considered to cause primarily PPAR*α*-dependent carcinogenicity in rodents, but it is considered to be relatively safe in humans, similar to other ligands [2]. However, Ward et al. [47] could not directly observe DEHP-derived tumors in the wild-type mice, because exposure to DEHP for 6 months may not be sufficient to induce hepatic tumors, as suggested by Marsman et al. [48]; they reported that DEHP tumorigenesis required longer exposure periods than Wy-14,643. It is doubtful whether DEHP definitively induces hepatic tumors via PPAR*α*.

As mentioned above, the following simple mechanism has been proposed for the DEHP-induced hepatocarcinogenesis; when DEHP was administered to rats and mice, the chemical caused an increase in cell proliferation and peroxisome proliferation [49]. The latter is accompanied by an increase in both peroxisomal and mitochondrial fatty acid metabolizing enzymes such as ACO. As a byproduct of fatty acid oxidation, enzymes involved with *β*-oxidation generate H_2_O_2_, resulting in elevated oxidative stress. DEHP also causes an increase in proinflammatory cytokines and inhibition of apoptosis [2, 24].

DEHP-induced liver carcinogenesis in rodents, however, appears to involve more complex pathways as described in the following events whereby various combinations of the molecular signals and multiple pathways may be involved [3]. DEHP is metabolized to bioactive metabolites which are absorbed and distributed throughout the body; they might induce PPAR*α*-independent activation of macrophages and production of oxidants, and also activate PPAR*α* and sustained induction of target genes. The inductions lead to enlargement of hepatocellular organelles, an increase in cell proliferation, a decrease in apoptosis, sustained hepatomegaly, chronic low-level oxidative stress and accumulation of DNA damage, and selective clonal expansion of the initiated cells. Finally, preneoplastic nodules might be induced and might result in adenomas and carcinoma.

Peraza et al. [10] also suggest that PPAR*α* is the only receptor in PPARs that is known to mediate carcinogenesis, while the prevailing evidence suggests that PPAR*β*, PPAR*γ*, and their ligands appear to be tumor modifiers that inhibit carcinogenesis, albeit there is still controversy in the field. Melnick [50] also addressed non-PPAR*α* mechanisms for DEHP-induced carcinogenicity as follows. (1) Peroxisome proliferator-induced tumorigenesis is related to the genes involved in cellular proliferations of, for example, p38 mitogen-activated protein kinase, which is not involved in peroxisome proliferations [51]. (2) DEHP and other peroxisome proliferators stimulated growth regulatory pathways such as immediate early genes for carcinogenesis (c-jun, c-fos, junB, egr-1), mitogen-activated protein kinase, extracellular signal-regulated kinase, and phosphorylation of p38, which were dissociated from PPAR*α* activation in rat primary cultures [52–54]. These findings also support the view that peroxisome proliferators, including DEHP, may have the potential for tumorigenesis via non-PPAR*α* signal pathways.

In recent years, an inflammation-associated model of cancers has been given attention [4, 5]. PPAR*α* exerts anti-inflammation effects by repressing nuclear factor kappa B (NF*κ*B) [55], which inhibits inflammation signaling and subsequent cancer [4].

Ito et al. [45] proposed possibility of DEHP tumorigenesis via a non-PPAR*α* pathway using PPAR*α*-null mice. They compared DEHP-induced tumorigenesis in wild-type and PPAR*α*-null mice treated for 22 months with diets containing 0, 0.01, or 0.05% DEHP. Surprisingly, the incidence of liver tumors was higher in PPAR*α*-null mice exposed to 0.05% DEHP (25.8%) than in similarly exposed wild-type mice (10%), while the incidence was 0% in wild-type miceand 4% in PPAR*α*-null mice without DEHP exposure. The levels of 8-hydroxydeoxyguanosine increased dose-dependently in mice of both genotypes, but the degree of increase was higher in PPAR*α*-null mice than in wild-type mice. NF*κ*B levels also significantly increased in a dose-dependent manner in PPAR*α*-null mice. The proto-oncogene c-jun-mRNA was induced, while c-fos-mRNA tended to be induced only in PPAR*α*-null mice fed with 0.05% DEHP-containing diet. These results suggest that chronic low-level oxidative stress induced by DEHP exposure may lead to the induction of inflammation and/or the expression of proto-oncogenes, resulting in a high incidence of tumorigenesis in PPAR*α*-null mice. Moderate activated PPAR*α* might protect from p65/p50 NF*κ*B inflammatory pathway caused by chronic DEHP exposure in wild-type mice. Although cross-talk of PPAR*γ*, but not PPAR*α*, with cyclooxygenase 2 (Cox-2), which also was related with inflammation-induced hepatocellular carcinoma, has been suggested [56], there was neither induction of Cox-2 nor PPAR*γ* in both genotyped mice of that study (data not shown).

Additionally, we compared the mechanisms of tumorigenesis between wild-type mice and PPAR*α*-null mice using hepatocellular adenoma tissues of both genotyped mice [57]. The microarray profiles showed that the up- or downregulated genes were quite different between hepatocellular adenoma tissues of wild-type mice and PPAR*α*-null mice exposed to DEHP, suggesting that their tumorigenesis mechanisms might be different. Interestingly, the gene expressions of apoptotic peptidase activating factor 1 and DNA-damage-inducible 45*α* (Gadd45*α*) were increased in the hepatocellular adenoma tissues of wild-type mice exposed to DEHP, whereas they were unchanged in corresponding tissues of PPAR*α*-null mice. On the other hand, the expressions of cyclin B2 and myeloid cell leukemia sequence 1 were increased only in the hepatocellular adenoma tissues of PPAR*α*-null mice. Taken together, DEHP may induce hepatocellular adenomas, partly via suppression of G2/M arrest regulated by Gadd45*α* and caspase 3-dependent apoptosis in PPAR*α*-null mice. However, these genes may not be involved in tumorigenesis in wild-type mice. In contrast, the expression level of Met was notably increased in the liver adenoma tissue of wild-type mice, which may suggest the involvement of Met in DEHP-induced tumorigenesis in wild-type mice. However, we could not determine whether DEHP promoted the spontaneous liver tumor in PPAR*α*-null mice because spontaneous hepatocellular tumors are known to occur in these mice at 24 months of age [58], while we observed DEHP-induced tumorigenesis at 22 months of age. To clarify this, gene expression profiles of liver tumors in the control group must be analyzed.

Taken together, the mechanisms of DEHP-induced carcinogenesis do not consist of only a simple pathway such as PPAR*α*-mediated peroxisome proliferation as mentioned by Rusyn et al. [3]. PPAR*α*-independent pathways may also exist and, by contrast, activated PPAR*α* may protect against DEHP-induced carcinogenesis. The valance of the production of oxidative stress via the transactivation of PPAR*α* and subsequent DNA damages versus the effective exertion of anti-inflammation by activating the receptor may determine the incidence of DEHP-induced tumors.

## 7. FUTURE INVESTIGATIONS

To determine the mechanism of species difference in response to peroxisome proliferators, a mouse line with human PPAR*α* was produced and designated hPPAR*α* ^TetOff^ [27]. This mouse line expresses the human receptor in liver in a PPAR*α*-null background by placing the hPPAR*α* cDNA under control of the Tet-Off system of doxycycline control with the liver-specific LAP1 (C/EBP*β*) promoter. Interestingly, the hPPAR*α* ^TetOff^ mice express the human PPAR*α* protein at levels comparable to those expressed in wild-type mice; so we should not need to consider the species differences in the expression of PPAR*α* between mice and humans. Treatment of this mouse line with Wy-14,643 revealed induction of genes' encoding peroxisomal lipid-metabolizing enzymes, including ACO, bifunctional enzyme and peroxisomal thiolase, and the fatty acid transporter CD36 at a level comparable to that in wild-type mice, expressing native mouse PPAR*α*. This suggested that human PPAR*α* is functionally active. Upon treatment with Wy-14,643, hPPAR*α* ^TetOff^ mice also had lower levels of fasting serum total triglycerides similar to wild-type mice. However, hPPAR*α* ^TetOff^ mice did not show any significant hepatocellular proliferation, nor did they have an induction of cell cycle control genes, in contrast to Wy-14,643-treated wild-type mice where a significant increase in mRNAs encoding PCNA, cMYC, cJUN, CDK1, CDK4, and several cyclins was found after treatment with Wy-14,643. hPPAR*α* ^TetOff^ mice were also found to be resistant to Wy-14,643-induced hepatocarcinogenesis after 11 months of Wy-14,643 feeding in contrast to a 100% incidence in the wild-type mouse group [59].

Another transgenic mouse line with human PPAR*α* was generated that has the complete human PPAR*α* gene on a P1 phageartificial chromosome (PAC) genomic clone, introduced onto the mouse PPAR*α*-null background [60]. This new line, designated hPPAR*α* ^PAC^, expresses human PPAR*α* not only in liver but also in kidney and heart. hPPAR*α* ^PAC^ mice exhibited responses similar to wild-type mice when treated with fenofibrate lowering of serum triglycerides and induction of PPAR*α* target genes' encoding enzymes involved in fatty acid metabolism. Treatment of hPPAR*α* ^PAC^ mice with fenofibrate did not cause significant hepatomegaly and hepatocyte proliferation similar to hPPAR*α* ^TetOff^ mice, suggesting that the resistance to the hepatocellular proliferation found in the hPPAR*α* ^TetOff^ mice is not due to lack of expression of the receptor in tissues other than liver.

Until now, there are no reports concerning the interaction between DEHP and hPPAR*α* ^TetOff^ or hPPAR*α* ^PAC^. Recently, we have compared the transactivation of mouse and human PPAR*α* by DEHP treatments using wild-type and hPPAR*α* ^TetOff^ mice (unpublished observation). A relatively high dose of DEHP (5 mmol/kg for 2 weeks) clearly activated PPAR*α* in liver of both genotyped mice, but the activation was very little in hPPAR*α* ^TetOff^ mice from the standpoint of the target gene expression as well as triglyceride levels in plasma and liver. Human PPAR*α* response to DEHP may be weak when sufficient human PPAR*α* is expressed in the human liver. Thus, the use of the hPPAR*α* ^TetOff^ mouse model is a very valuable means to solve the species differences in the toxicity of peroxisome proliferators. The results from the typical peroxisome proliferator (Wy-14643) may not always be similar to those of DEHP; a study of each case is needed using hPPAR*α* ^TetOff^ mouse model.

## 8. PROPOSED SCIENCE POLICY STATEMENTS

The International Agency for Research on Cancer downgraded the level of potential health risks of DEHP from 2b (possibly carcinogenic to humans) to 3 (not classifiable as to carcinogenicity to humans) in 2000 [61]. In this report, DEHP carcinogenesis via PPAR*α* was considered not to be relevant to humans because peroxisome proliferation had not been documented either in human hepatocyte cultures exposed to DEHP or in the liver of nonhuman primates. This decision has been variously argued by several scientists in the literature [50, 62, 63]. In contrast, the Japan Society for Occupational Health has maintained the 2B class of DEHP carcinogenicity because of the obvious rodent carcinogenicity [64].

Although the US Environmental Protection Agency (EPA) had classified the risk for DEHP carcinogenicity as B2 (probable human carcinogen) in 1993, recently, the expert panel of EPA report has provided the current scientific understanding of the mode(s) of action of PPAR*α* agonist-induced tumors observed in rodent bioassays that are associated with PPAR*α* agonisms: liver tumors in rats and mice as well as Leydig cell and pancreatic acinar cell tumors in rats—all of which represent limited evidence [65]. Since the key events for the mode of action, which have been causally related to liver tumor formation, include the activation of PPAR*α*, perturbation of cell proliferation and apoptosis, selective clonal expansion, and the PPAR*α*-related key events included in the expression of peroxisomal genes (e.g., palmitoyl CoA oxidase and acyl CoA oxidase) and peroxisome proliferation (i.e., an increase in the number and size of peroxisomes) are reliable markers. Additionally, the evidence obtained from the findings that PPARa agonists did not activate the receptor in human cell culture or biopsy samples, and from epidemiological studies, shows that humans are apparently refractory to the effects of a PPAR*α* agonist. However, the EPA maintained the DEHP carcinogenicity criterion.

In 2004, with regard to preclinical and clinical safety assessments for PPAR agonists, the Food and Drug Administration recommended that, due to the prevalence of positive tumor findings of PPAR agonists, two-year carcinogenicity studies on mice and rats are required [66].

Although IARC changed the criterion for DEHP carcinogenicity, other agencies did not because DEHP is a potential rodent carcinogen of liver and the precise mechanism has not been yet understood, though DEHP is a potentially hepatic carcinogen in rodents.

## 9. CONCLUSIONS

As mentioned above, some studies suggest the possibility of DEHP tumorigenesis via a non-PPAR*α* pathway although DEHP also exerts adverse effects via PPAR*α*-dependent pathway. Since there are species differences regarding expression levels, cellular context, and function of PPAR*α* as well as metabolism enzyme activity of DEHP, it is difficult to extrapolate the results from rodents to humans in terms of risk. Recently, hPPAR*α* mice have been developed, which may help to solve these differences. Re-evaluation of the risk of DEHP carcinogenicity may well be warranted if the previous decisions were based on only PPAR*α*-dependent mechanisms.

## Figures and Tables

**Table 1:** Primary studies on DEHP-induced carcinogenesis in mice and rats (modifying the paper reported by Huber et al. [2]).

Author	Sex	Route	Duration	Dosage	Type of tumor	Tumor frequency (%)	
Species, strain							
[39] Rat F344	M	Feed	103 w	0.00%	Hepatic tumors	6	
				0.60%		12	
				1.20%		24	
	F	Feed	103 w	0.00%	Hepatic tumors	0	
				0.60%		12	
				1.20%		26	
[41] Rat F344	F	Feed	2 y	0.00%	Hepatic tumors	0	
				0.03%		6	
				0.10%		5	
				1.20%		30	
[42] Rat F344	M	Oral	24 m	0 (water)	Liver carcinoma	4	
				0 (vehicle)		12	
				2EH 50 mg/kg		6	
				2EH 150		6	
				2EH 500		2	
	M	Oral	24 m	0 (water)	Liver adenoma	0	
				0 (vehicle)		0	
				2EH 50 mg/kg		0	
				2EH 150		2	
				2EH 500		0	
	F	Oral	24 m	0 (water)	Liver carcinoma	0	
				0 (vehicle)		2	
				2EH 50 mg/kg		2	
				2EH 150		4	
				2EH 500		0	
[42] Mouse B6C3F1	M	Oral	18 m	0 (water)	Liver carcinoma	8	
				0 (vehicle)		12	
				2EH 50 mg/kg		12	
				2EH 200		14	
				2EH 750		18	
	M	Oral	18 m	0 (water)	Liver adenoma	0	
				0 (vehicle)		0	
				2EH 50 mg/kg		0	
				2EH 200		0	
				2EH 750		2	
	F	Oral	18 m	0 (water)	Liver carcinoma	2	
				0 (vehicle)		0	
				2EH 50 mg/kg		2	
				2EH 200		6	
				2EH 750		10	
[43] Rat F344	M	Feed	2 y	0 ppm	Hepatocellular carcinoma	2	
				6000 ppm		2	
				12000 ppm		10	
	M	Feed	2 y	0 ppm	Hepatocellular neoplastic nodule	4	
				6000 ppm		10	
				12000 ppm		14	
	F	Feed	2 y	0 ppm	Hepatocellular carcinoma	0	
				6000 ppm		4	
				12000 ppm		16	
				0 ppm	Hepatocellular neoplastic nodule	0	
				6000 ppm		8	
				12000 ppm		10	
[43] Mouse B6C3F1	M	Feed	2 y	0 ppm	Hepatocellular carcinoma	18	
				3000 ppm		29	
				6000 ppm		38	
				0 ppm	Hepatocellular adenoma	10	
				3000 ppm		23	
				6000 ppm		20	
	F	Feed	2 y	0 ppm	Hepatocellular carcinoma	0	
				3000 ppm		14	
				6000 ppm		34	
				0 ppm	Hepatocellular adenoma	2	
				3000 ppm		10	
				6000 ppm		2	
[40] Rat F344	M	Diet	79 w	0 ppm	Hepatocellular carcinoma	10	
				2500 ppm		0	
				12500 ppm		40	
				0 ppm	Hepatocellular adenoma	10	
				2500 ppm		10	
				12500 ppm		10	
	F	Diet	79 w	0 ppm	Hepatocellular carcinoma	0	
				2500 ppm		0	
				12500 ppm		20	
				0 ppm	Hepatocellular adenoma	0	
				2500 ppm		0	
				12500 ppm		10	
	M	Diet	105 w	0 ppm	Hepatocellular carcinoma	1	
				100 ppm		0	
				500 ppm		2	
				2500 ppm		5	
				12500 ppm		30	
				Recovery		13	
				0 ppm	Hepatocellular adenoma	5	
				100 ppm		10	
				500 ppm		5	
				2500 ppm		12	
				12500 ppm		26	
				Recovery		22	
				0 ppm	Hepatocellular carcinoma	0	
				100 ppm		2	
				500 ppm		0	
				2500 ppm		2	
				12500 ppm		18	
				Recovery		7	
	F	Diet	105 w	0 ppm	Hepatocellular adenoma	0	
				100 ppm		6	
				500 ppm		2	
				2500 ppm		3	
				12500 ppm		10	
[40] Mouse B6C3F1	M	Diet	79 w	0 ppm	Hepatocellular carcinoma	0	
				100 ppm		0	
				500 ppm		10	
				1500 ppm		0	
				6000 ppm		7	
				0 ppm	Hepatocellular adenoma	7	
				100 ppm		10	
				500 ppm		20	
				1500 ppm		10	
				6000 ppm		7	
	F	Diet	79 w	0 ppm	Hepatocellular carcinoma	0	
				100 ppm		0	
				500 ppm		0	
				1500 ppm		0	
				6000 ppm		13	
				0 ppm	Hepatocellular adenoma	0	
				100 ppm		10	
				500 ppm		10	
				1500 ppm		10	
				6000 ppm		27	
	M	Diet	105 w	0 ppm	Hepatocellular carcinoma	6	
				100 ppm		8	
				500 ppm		14	
				1500 ppm		22	
				6000 ppm		31	
				Recovery		22	
				0 ppm	Hepatocellular adenoma	6	
				100 ppm		17	
				500 ppm		20	
				1500 ppm		22	
				6000 ppm		27	
				Recovery		5	
	F	Diet	105 w	0 ppm	Hepatocellular carcinoma	4	
				100 ppm		3	
				500 ppm		5	
				1500 ppm		15	
				6000 ppm		23	
				Recovery		42	
				0 ppm	Hepatocellular adenoma	0	
				100 ppm		3	
				500 ppm		6	
				1500 ppm		14	
				6000 ppm		49	
				Recovery		24	
[43] Rat F344	M	Feed	2 y	0, 6000, 12000 ppm	Pituitary adenoma or carcinoma	Decrease in highest dose	
	F	Feed	2 y	0, 6000, 12000 ppm	Pituitary adenoma or carcinoma	Decrease in lower dose	
	M	Feed	2 y	0, 6000, 12000 ppm	Thyroid C-cell adenoma or carcinoma	Decrease in highest dose (unclear)	
	M	Feed	2 y	0, 6000, 12000 ppm	Testis interstitial cells tumor	Decrease in highest dose	
	F	Feed	2 y	0, 6000, 12000 ppm	Mammary gland	Decrease in highest dose	
[36] Rat F344	M	Diet	78 w	0 ppm	Interstitial cells tumor or testes	90	
				2500 ppm		100	
				12500 ppm		30	
	M	Diet	104 w	0 ppm	Interstitial cells tumor or testes	92	
				100 ppm		90	
				500 ppm		91	
				2500 ppm		92	
				12500 ppm		31	
				0 ppm	Mononuclear cell leukemia	23	
				100 ppm		26	
				500 ppm		29	
				2500 ppm		49	
				12500 ppm		42	
				0 ppm	Pancreatic acinar cell adenoma	0	
				100 ppm		0	
				500 ppm		0	
				2500 ppm		0	
				12500 ppm		8	
	F	Diet	104 w	0 ppm	Mononuclear cell leukemia	22	
				100 ppm		34	
				500 ppm		20	
				2500 ppm		25	
				12500 ppm		26	
				0 ppm	Pancreatic acinar cell adenoma	0	
				100 ppm		0	
				500 ppm		0	
				2500 ppm		0	
				12500 ppm		3	
[44] Rat F344	M	Diet	79 w	0 ppm	Interstitial cells tumor or testes	90	
				12500 ppm		30	
				0 ppm	Mononuclear cell leukemia	0	
				12500 ppm		10	
	M	Diet	105 w	0 ppm	Interstitial cells tumor or testes	92	
				12500 ppm		31	
				Recovery		32	
				0 ppm	Mononuclear cell leukemia	23	
				12500 ppm		42	
				Recovery		53	
[35] Mouse B6C3F1	M,	Diet	78 w,	0, 100, 500, 1500, 6000 ppm	No data about tumors		
[44] Rat F344	F	Diet	79 w	0 ppm, 12500 ppm	No data about tumors		
						
[44] Mouse B6C3F1	M,	Diet	79 w	0 ppm, 6000 ppm,	No data about tumors		
	M,	Diet	105 w	0 ppm, 6000 ppm,	No data about tumors		
[45] Mouse 129/Sv, PPAR*α*-null	M	Diet	21 m		Liver tumors (hepatocellular adenoma, hepatocellular carcinoma, cholangiocellular carcinoma)	Wild-type	PPAR*α*-null
				0%		0	4
				0.01%		9	4
				0.05%		10	25.8
